# Suite of simple metrics reveals common movement syndromes across vertebrate taxa

**DOI:** 10.1186/s40462-017-0104-2

**Published:** 2017-06-01

**Authors:** Briana Abrahms, Dana P. Seidel, Eric Dougherty, Elliott L. Hazen, Steven J. Bograd, Alan M. Wilson, J. Weldon McNutt, Daniel P. Costa, Stephen Blake, Justin S. Brashares, Wayne M. Getz

**Affiliations:** 1 NOAA Southwest Fisheries Science Center, Environmental Research Division, 99 Pacific Street, Monterey, CA 93940 USA; 2 Department of Environmental Science, Policy, and Management, University of California, Berkeley, CA 94720 USA; 3 Department of Ecology and Evolutionary Biology, University of California, Santa Cruz, CA 95060 USA; 4 Structure & Motion Lab, Royal Veterinary College, University of London, London, UK; 5 Botswana Predator Conservation Trust, Maun, Botswana; 6 Max Planck Institute for Ornithology, Radolfzell, Germany; 7 School of Mathematical Sciences, University of KwaZulu-Natal, Durban, South Africa

**Keywords:** Movement ecology, Migration, Nomadism, Central place foraging, Territoriality, GPS data, Classification scheme, Cluster analysis

## Abstract

**Background:**

Because empirical studies of animal movement are most-often site- and species-specific, we lack understanding of the level of consistency in movement patterns across diverse taxa, as well as a framework for quantitatively classifying movement patterns. We aim to address this gap by determining the extent to which statistical signatures of animal movement patterns recur across ecological systems. We assessed a suite of movement metrics derived from GPS trajectories of thirteen marine and terrestrial vertebrate species spanning three taxonomic classes, orders of magnitude in body size, and modes of movement (swimming, flying, walking). Using these metrics, we performed a principal components analysis and cluster analysis to determine if individuals organized into statistically distinct clusters. Finally, to identify and interpret commonalities within clusters, we compared them to computer-simulated idealized *movement syndromes* representing suites of correlated movement traits observed across taxa (migration, nomadism, territoriality, and central place foraging).

**Results:**

Two principal components explained 70% of the variance among the movement metrics we evaluated across the thirteen species, and were used for the cluster analysis. The resulting analysis revealed four statistically distinct clusters. All simulated individuals of each idealized movement syndrome organized into separate clusters, suggesting that the four clusters are explained by common movement syndrome.

**Conclusions:**

Our results offer early indication of widespread recurrent patterns in movement ecology that have consistent statistical signatures, regardless of taxon, body size, mode of movement, or environment. We further show that a simple set of metrics can be used to classify broad-scale movement patterns in disparate vertebrate taxa. Our comparative approach provides a general framework for quantifying and classifying animal movements, and facilitates new inquiries into relationships between movement syndromes and other ecological processes.

**Electronic supplementary material:**

The online version of this article (doi:10.1186/s40462-017-0104-2) contains supplementary material, which is available to authorized users.

## Background

Animal movement is an essential determinant of individual fitness (e.g. resource acquisition, survival), with critical implications for population persistence (e.g. dispersal, gene flow), species distributions, and ecosystem function (e.g. ecosystem engineering, propagule dispersal) [1–4]. In the so-called Anthropocene, movement will also play a critical role in species and community responses to environmental change [5–7]. Because of the profound importance of movement in driving the spatial dynamics of multiple levels of ecological organization, a deeper integration of movement ecology into wildlife ecology and conservation biology has recently been highlighted as a research priority [8]. A rigorous classification of movement patterns is needed, independent of the mechanisms that produce those patterns, to better link movement ecology with other areas of ecological research. In particular, these patterns may be used to inform predictions in such areas as the invasive potential of exotic species [9], how diseases may spread through contact-networks [10], or how species may respond to climate change [11]. Pattern classification tools will moreover allow us to investigate factors, including life history traits, that produce common movement patterns across organisms. Yet, because empirical studies of animal movement are most-often site- and species-specific [12], we lack a sense of the extent to which classes of movement patterns recur across diverse organisms.

In behavioral ecology, the concept of behavioral syndromes, i.e. suites of correlated behaviors, has aided quantification of animal behavioral types and their integration into ecological and evolutionary studies [13]*.* Analogously, we aim to quantitatively characterize *movement syndromes*, i.e. suites of correlated movement traits [14], such as migration or nomadism [13, 15]. A rapidly growing body of movement studies has generated a number of promising methods and metrics to differentiate movement patterns [16–18], but few empirical studies have tested the utility of these metrics across multiple species, let alone highly diverse vertebrate taxa [19]. Different taxa not only have different modes of movement (e.g., swimming versus terrestrial locomotion), but also move across spatial and temporal scales that differ by orders of magnitude. Thus, a unified framework for characterizing movement syndromes requires an examination across a range of taxa, movement modes, and body sizes.

Here, we provide the some of the first empirical evidence that statistical signatures of animal movement patterns recur across widely disparate taxa and can be used to classify movement syndromes for terrestrial, aerial and marine species. Three movement syndromes appear repeatedly in the literature from which we draw upon: range residency, nomadism, and migration [17, 19–21]. Range residency can be further expanded upon to include central place foraging and territoriality, yielding four movement syndromes classically defined as: 1) central-place foraging, in which individuals return to fixed locations between foraging trips [22]; 2) territoriality, in which individuals actively demarcate the boundaries of fixed areas against conspecifics [23]; 3) nomadism, in which individuals move unpredictably with little to no site fidelity [24]; 4) migration, in which individuals move with persistence from one habitat area to another, bi-directionally and with temporal predictability [25]. These movement syndromes may be lifetime descriptors correlated with life history types, or life history stage descriptors of significant movement phases (e.g. breeding, resource pulses, etc.). We excluded dispersal from our assessment because it is a rare, life-history-related event that typically occurs over short time-scales [26]. While the four syndromes considered in our study differ conceptually and qualitatively, we develop a novel methodology for their quantitative distinction.

We expected similar forms and characteristics of movement to underlie the same syndrome across taxa, movement modes, and body sizes. To evaluate this prediction and test whether simple metrics can reliably classify movement syndromes, we assessed five key movement metrics for GPS trajectories of individuals from thirteen species spanning three taxonomic classes, continents, movement modes, and orders of magnitude in body size. Using these metrics, we performed a cluster analysis to determine if our study organisms fell into statistically distinct groupings. We compared the resulting four groupings with simulations of the four idealized syndromes – central place foraging, territoriality, nomadism, and migration – which revealed that observed groupings were explained by common movement syndromes. Thus, our approach provides a framework for a rigorous large-scale movement classification scheme that may facilitate the integration of animal movement into other areas of ecology by pairing the movement syndrome of an animal with ecological and life-history data to develop and test predictions.

## Methods

### Empirical data

We gathered satellite-derived movement data for the following species: African buffalo (*Syncerus caffer*), African elephant (*Loxodonta africana*), African wild dog (*Lycaon pictus*), black-backed jackal (*Canis mesomelas*), California sea lion (*Zalophus californianus*), cheetah (*Acinonyx jubatus*), Galapagos albatross (*Phoebastria irrorata*), Galapagos tortoise (*Geochelone nigra*), African lion (*Panthera leo*), northern elephant seal (*Mirounga angustirostris*), plains zebra (*Equus quagga*), springbok (*Antidorcas marsupialis*), and white-backed vulture (*Gyps africanus*). Species were chosen to represent an array of taxa, environments, and body sizes, but were restricted by the availability of datasets with sufficient quality in terms of resolution (<= 1 h fix intervals) and duration (continuous data collection for > = 2 months to allow for quantification of monthly home range overlap), with the exception of Galapagos albatross data that were collected at 90-min intervals. All datasets were derived from GPS units except the California sea lion and northern elephant seals that were fitted with ARGOS tags; for these, data were first filtered for errors and smoothed using a state space model to obtain hourly position estimates using the R package *crawl* [27–30]. All data were resampled to a 1-h resolution to achieve consistent fix rates for comparison. To check the sensitivity of our results to temporal resolution, we reran the following analyses at 3-h intervals. See Additional file 1: Table S1 for a detailed summary of data and sources.

### Movement metrics

We employed five metrics widely applied in current movement studies and grounded in ecological theories of animal movement in heterogeneous landscapes. Turn angle correlation and net-squared displacement are two central parameters in random-walk (RW) models, which are extensively used to evaluate animal search strategies and foraging efficiency [30–33]. Variations of random walk models have been shown to approximate nomadic movement via uncorrelated RWs [17], central-place foraging via biased RWs [34], and territorial behavior via correlated RWs [35]. When spatially-explicit information about the landscape is known, ecologists have employed a variety of time-use metrics to quantify how animals exploit resources. In heterogeneous landscapes, for example, animals are predicted to adjust their residence times and/or return times to a given area in response to variation in resource quality [36–38]; these two properties have been linked to emerging patterns of home range residency [38]. Over longer timescales, measures of home range stability, such as the amount of overlap between seasonal home ranges, can inform theory on how animals respond to temporal predictability of resources [39] and have been used to identify migration patterns [40]. Because movement processes are often scale-dependent and those of a given syndrome may be observable at one or more spatiotemporal scales [41], we considered that our metrics were relevant over a range of timescales — in our case, hour, day, month, and lifetime of trajectory. Thus, for each individual in our dataset, we calculated five movement metrics suitable for analysis over these timescales as follows:

1. *Mean turn angle correlation* (TAC). Following Dray et al. (2010), we calculated angular autocorrelation *S*_*A*_ as the sum of squares of chord distances between N successive turn angles *ρ*:

$$ {S}_A=\frac{1}{N}\ \sum_{n=1}^{N-1}\left[{\left(\mathit{\cos}{\rho}_{n+1} - \mathit{\cos}{\rho}_n\right)}^2+{\left(\mathit{\sin}{\rho}_{n+1} - \mathit{\sin}{\rho}_n\right)}^2\right] $$

Thus, small chord distances resulting in low *S*_*A*_ values correspond to high turn angle correlation [42].

2. *Mean residence time* (RT). Residence time was measured as the number of hours the animal spends inside a circle of a given radius centered on each location without leaving the radius for more than a specified cut-off time [38]. We tested the sensitivity of a subset of our dataset to radii of mean step length (SL), 2 x mean SL, 4 x mean SL, and 8 x mean SL, where SL was calculated as the mean Euclidean distance between successive relocations, and cut-off times of 12 and 24 h. Consistent time-use patterns were observed across these thresholds, so following van Moorter et al. (2015), we used a radius of mean SL and a 12-h cut-off time.

3. *Mean time-to-return* (T2R). Time-to-return was measured as the number of hours the animal spends beyond a specified cut-off time before its return to a circle of a given radius centered on each location [38]. We conducted the same sensitivity analysis for this metric as above, and finding consistent patterns across thresholds, we again used a radius of mean SL and a 12-h cut-off time.

4. *Mean volume of intersection* (VI). Volume of intersection was measured by the overlap between monthly 95% kernel density home ranges [43, 44]. Volume of intersection varies between 0 and 1, with increasing values corresponding to increasing overlap between monthly home ranges. VI is thus a measure of home range stability.
5. *Maximum net squared displacement* (MNSD). Maximum net squared displacement was calculated as the maximum squared Euclidean displacement from the first relocation of the trajectory over the full course of the trajectory [45]. To make comparisons among individuals across species that have orders of magnitude different motion capacities, we scaled this parameter for each individual by dividing by the smallest MNSD observed for its species.

All movement metrics were calculated using the *adehabitatLT* and *adehabitatHR* packages [46, 47] in R 3.3.2 [29].

### Cluster analysis

To elucidate any underlying structure in our dataset, we performed a principal components analysis (PCA) for the above metrics calculated from our empirical datasets using the *prcomp* function in the R *stats* library [29]. PCA is a widely used technique for summarizing a multivariate dataset into a reduced number of uncorrelated dimensions, or *principal components*, while minimizing the loss of information in the original dataset [48]. We used the Broken-stick criterion to retain important composite (PC) axes, in which components are retained if their eigenvalues exceed those expected by random data [43]. Comparative analyses of component retention methods have shown the Broken-stick model to be among the most reliable techniques [48, 49]. To normalize the dataset for this analysis we first log-transformed the data, followed by centering around the mean and dividing by the variance [50].

Finally, we applied Ward’s agglomerative hierarchical clustering algorithm to the resulting PCA values [51] using the *hclust* function in the R *stats* library [29]. This approach clusters the most similar pair of points based on their squared Euclidean distance at each stage of the algorithm, and is an efficient method to identify clusters based on minimum within-cluster variance without making an a priori determination of the number of clusters to generate [52]. These clusters can be viewed as functional movement groups, analogous to functional types first theorized for plant communities, which provide a non-phylogenetic classification based on shared responses to environmental conditions [53]. To determine the significance of the resulting cluster arrangement, we calculated *p*-values for each cluster via multi-scale bootstrap resampling with 1000 bootstrap replications using the R package *Pvclust* [54, 55]. By simulating the following idealized movers and determining their cluster assignments, we were able to identify these clusters by movement syndrome.

### Simulated data

**Fig. 1:**
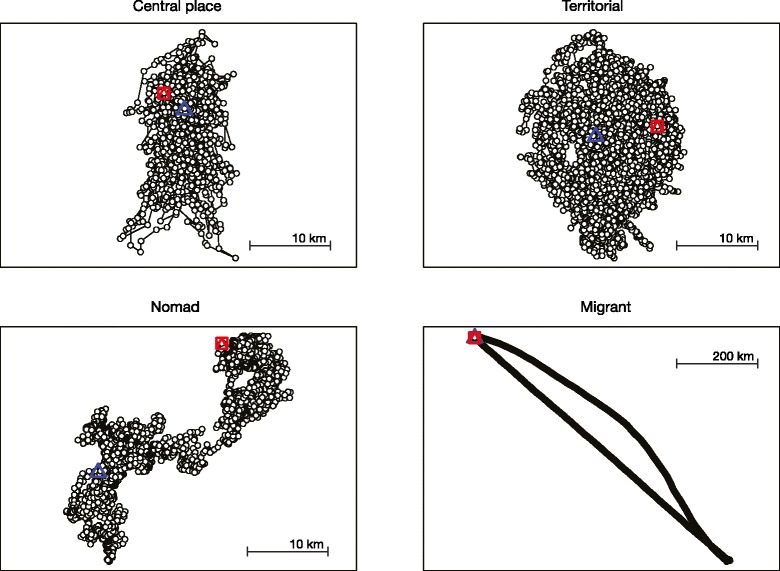
Sample path simulations for four idealized movement syndromes. Movement paths begin at the blue triangle and end at the red square

To interpret the resulting clusters, we developed spatially-explicit models simulating four movement syndromes: central place foraging, territorial, nomadic, and migratory (Fig. 1). Central place foragers and territorial individuals were assumed to have stable home ranges, whereas nomadic and migratory individuals moved without boundary constraints. For each syndrome, we simulated six individuals, using rules described below. In all cases, we drew step length and turning angle randomly from probability distributions, enabling variation in the movement paths of individuals within the same syndrome. We simulated data for each individual for 3600 time steps at hourly intervals, with the exception of migratory individuals, which we simulated for 7200 time steps to incorporate a return migration. R code for these simulations is provided in Additional file 2, with additional information about parameter settings including scalar multipliers for step size and standard deviations of normal distributions used for correlated turning angles.

#### Central Place Foragers (CPFs)

For CPFs, we assumed that resources are optimal at the center of the home range (the ‘central place’) [56]. We drew steps within the core of the home range from a uniform distribution and weighted them by the distance to the edge of the home range to simulate greater space use within the core. Upon reaching the home range boundary, we drew turning angles from a normal distribution with a mean 180**°** from the direction the simulated individual was traveling rather than a uniform distribution, leading to its return to the home range center.

#### Territorialists

The territorial individual functioned in an opposite fashion from CPFs in terms of its selective use of the outer edges of its home range – in effect demarcating or defending the territory [57]. Steps were weighted by the distance to the home-range center. Turning angles, however, were adjusted as for CPFs to maintain home range stability.

#### Nomads

We assigned these individuals randomly to one of two states: foraging or exploratory [58]. The probability of switching from one state to the other in nomads was 0.05 based on empirical estimates ranging from 0.018–0.09 [59, 60]. The foraging state was meant to simulate movement patterns in the vicinity of high quality resources, so we applied lower weights to step sizes for the foraging state than for the exploratory state. We drew turning angles from a uniform distribution for the foraging state and a normal distribution for the exploratory state with a mean of the initial direction after switching from the foraging state.

#### Migrants

We assigned these individuals to one of two states: sedentary or migratory [58]. In the sedentary state, we defined movement by uniform step size and turning angle distributions. We defined the migratory state by highly directional movement, with long step sizes and highly correlated turning angles [61]. After an approximately four-month period of residence, the individuals migrated for about two months before entering a sedentary state for another four months at their new location, then returned to their origin location over the course of a two month return migration.

## Results

**Fig. 2:**
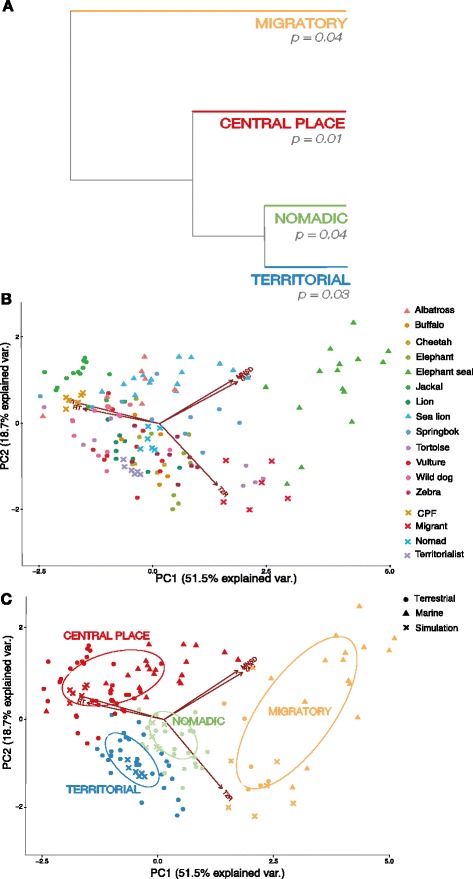
**a** Dendrogram tree displaying results of Ward hierarchical cluster analysis of all individuals based on PC1 and PC2 values, and bootstrapped *p*-values for each cluster. See Additional file 3: Figure S1 for full display of individual leaves within each major cluster. **b** Scatterplot of individuals based on PCA-defined axes. Simulated individuals are plotted for reference, although not included in the PCA. **c** Scatterplot of classified individuals based on PCA-defined axes. Ellipses represent the 50% probability contour for cluster classifications

**Fig. 3:**
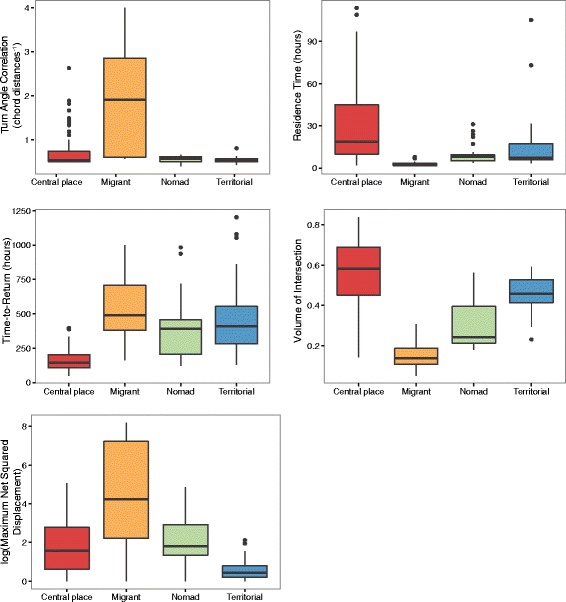
Boxplots of movement metrics for syndrome classifications, excluding simulated individuals

The first two principal components (PC) of the PCA explained 70% of the variance among the five movement metrics and thus PC1 and PC2 were retained for the cluster analysis using the Broken-stick criterion (Table 1). Plotting our data along the minor PC axes (PCs 3, 4, and 5) did not provide informative clusters, suggesting that the first two PCs are sufficient for classifying individuals by syndrome (Additional file 3: Figure S2). Using acronyms VI (Volume of Intersection), RT (Residence Time), T2R (Time-to-Return), TAC (Turn Angle Correlation) and MNSD (Maximum Net Squared Displacement), the first PC represented a contrast primarily between VI + RT and TAC + MNSD, with a somewhat smaller contribution of T2R, to the latter. The second PC represented a contrast primarily between T2R and TAC + MNSD. Because of evidence of collinearity between MNSD and TAC, as well as VI and RT (Fig. 2b), we ran the PCA using different combinations of a reduced set of three variables (Additional file 3: Figure S3). Despite potential collinearity, we found that including only three variables performed less well than including all five, presumably because some variables play a larger role in classifying particular syndromes than others (Fig. 3).

**Table 1:** Contributions of variables to and cumulative percentage of variance explained by principal components. PC1 and PC2 are significant components based on the Broken-stick criterion and retained for the cluster analysis

	PC1	PC2	PC3	PC4	PC5
Turn Angle Correlation	0.47	0.47	−0.12	−0.55	−0.50
Residence Time	−0.46	0.17	0.72	0.04	−0.50
Time-to-Return	0.35	−0.68	0.46	−0.45	0.08
Volume of Intersection	−0.50	0.23	−0.00	−0.67	0.49
Maximum Net Squared Displacement	0.44	0.48	0.51	0.21	0.51
Cumulative Percentage of Variance Explained	51.5%	70.1%	84.4%	94.8%	100%

The resulting cluster analysis identified four statistically significant groupings (Fig. 2a). All individuals of the four simulated movement syndromes fell into separate groups. These results were robust to reducing the sampling resolution from hourly to 3-hourly (Additional file 4). A full dendrogram displaying individual leaves within clusters is provided in Additional file 3: Figure S1. The heights of the associated dendrogram branches correspond to the squared Euclidean distances between clusters in PCA-defined movement ecology space (Fig. 2b). Thus, clusters that diverge at lower heights (have shorter branches) have greater similarity. Multiple species were represented in more than one cluster, showing that more than one movement syndrome may occur within a monospecific population (Table 2; Additional file 3: Figure S1).

**Table 2:** Summary of 130 individuals within 13 species analyzed into cluster classifications

*Species*	*N individuals*	Migratory	Central place	Nomadic	Territorial
African buffalo	5	-	-	2	3
African elephant	8	-	1	4	3
African wild dog	13	-	9	1	3
Black-backed jackal	15	-	15	-	-
California sea lion	15	1	14	-	-
Cheetah	5	-	-	-	5
Galapagos albatross	8	-	8	-	-
Galapagos tortoise	8	4	4	-	-
Lion	9	-	1	1	7
N. elephant seal	15	15	-	-	-
Plains zebra	9	-	-	6	3
Springbok	10	2	4	4	-
White-backed vulture	10	-	2	3	5

Analysis of the movement metrics for each cluster revealed distinct differences between putative syndromes (Fig. 3). Specifically, individuals in the migrant cluster had the highest average turn angle correlation, times to return, and maximum net squared displacement, and the lowest average residence times and volume of intersection. In contrast, individuals in the central place cluster had the highest average RT and VI and lowest T2R. Individuals in the territorial cluster had next-to-highest T2R and VI, and lowest MNSD. Finally, individuals in the nomadic cluster had intermediate values for all metrics.

## Discussion

Quantitative descriptions of broad-scale movement patterns seen across taxa are limited by the species-specific nature of movement studies, often due to constraints in funding or feasibility [12]. By combining movement data from thirteen taxonomically diverse species with simulated movement trajectories representing four movement syndromes, our results offer an early indication of widespread recurrent patterns in movement ecology that have consistent statistical signatures, even in highly disparate ecological systems. Our analysis found that movement syndromes were conserved across ecotype, including marine systems which change more rapidly than terrestrial habitats [62]. We show that the trajectories of individuals can be classified into these movement syndromes using a suite of simple metrics. Ultimately, classifying individuals by movement syndromes provides a window to predicting spatial and broader life history patterns.

Importantly, our movement syndrome classifications did not simply divide by species membership, but instead indicated movement strategies common across individuals within their syndrome cluster. For some species, such as the black-backed jackal, all individuals were assigned to the same syndrome (Table 2). For other species, assignments were made to more than one syndrome. For instance, half of the Galapagos tortoises in our dataset exhibited seasonal altitudinal migrations [63] and were appropriately classified as migrants, while the remaining resident tortoises were classified as CPFs (see Additional file **3**: Figure S4 for movement paths). This highlights the value of examining intraspecific variability in movement behavior when assessing population-level movement patterns. It also emphasizes the need to consider the degree to which populations of a species contain multiple movement syndromes, particularly when developing conservation and management plans.

Results from our principal components analysis showed that movement syndromes could be differentiated based on two orthogonal axes. From left to right along PC1 were clustered CPFs/territorial individuals, nomads, and migrants (Fig. 2b), suggesting this axis indicates a spectrum of random walk movement from diffusive (low directional persistence) to advective (high directional persistence) movement [30]. In contrast, along PC2 territorial individuals have low values and CPFs have high values. This second axis can be interpreted as a continuum of low to high repeated use of resources, as territorial animals may limit returns to previously visited sites in exchange for patrolling a greater proportion of their territory [57], while CPFs by definition have high site fidelity and return rates to their ‘central place’ [56]. Contrary to our expectations, territorial individuals were more closely associated with nomads than CPFs (Fig. 2a), likely because of the strong role their similar values of T2R play in defining PC2 (Fig. 3). There is also a clear trend along the PC2 axis differentiating terrestrial species and marine species. Across movement syndromes, marine species — here, migratory Northern elephant seals and central placing foraging Galapagos albatrosses and California sea lions — have lower mean T2R and higher MNSD and TAC than their terrestrial counterparts. We hypothesize that these differences could be attributed to the high motion capacity of marine organisms [64], facilitation of movement in air and water with few static barriers requiring circumnavigation (resulting in higher turn angle correlation) [65], and greater dispersion of resources in pelagic environments [66].

No single metric could be used to distinguish the four movement syndromes, suggesting that these metrics must be assessed in concert. While significant headway has been made applying a single statistic such as Net Squared Displacement to differentiate between sedentary home range behavior, migration and nomadism in a single taxon [17, 21, 67], distinguishing between more complex forms of sedentary behavior such as territoriality versus central place foraging, and among disparate taxa, is a greater challenge. Thus, we recommend evaluating movement with multiple metrics in order to capture metric- or scale-dependent patterns. Our choices of metrics reflect those prevalent in current studies of movement ecology and were selected to represent multiple time scales of analysis relevant to resource use on land- and seascapes. Our results indicate that the metrics used here can serve as informative synoptic measures to classify a broad array of organisms into movement syndromes. However, future research should test the utility of other movement metrics in classifying organisms into additional meaningful classes in ecology. We also note that while our syndromes were defined by individual movement metrics, syndromes emerging from behavioral responses to conspecifics, such as territoriality, also could potentially be elucidated by evaluating movement patterns at a population level.

Additionally, many movement metrics will be sensitive to the temporal resolution or duration of the data collected [41, 68]. For the purposes of illustration, we investigated 1-h and 3-h resolution data collected for at least two months to allow quantification of monthly volume of intersection, yet other analyses may use different timescales that, for example, better capture single life events such as dispersal. Lower sampling rates have also been shown to reduce ability to distinguish among behavioral modes [69]. Indeed, our re-analysis for data subsampled to 3-h resolution had a harder time distinguishing between nomadic and central place foraging syndromes for some individuals (Additional file 4). Generally, metrics measuring movement at finer scales were more sensitive to the fix rate. For subsampled data, residence times increased and times to returns decreased when compared to those derived from hourly fixes. As demonstrated in other studies, turn angles became also less correlated at lower resolution [69], while volume of intersection and maximum net squared displacement metrics remained stable. Despite these differences, our analysis of lower resolution data retained four distinct clusters that matched the proposed syndromes in the simulations, and maintained the majority of syndrome assignments for the empirical data (Additional file 4).

While we cannot ground-truth the classification of each study animal in our dataset, their assignments are consistent with how we understand their movement processes, such as the tortoise assignments described above [63], northern elephant seals performing long-distance migrations [28], and California sea lions making repeated foraging trips from their breeding colony [70]. A priori predictions for individual African wild dogs, lions and cheetahs based on behavioral observations made during movement data collection also match their classifications (Botswana Predator Conservation Trust, *personal communication*). Because the classification scheme is determined by our syndrome simulations, assignments may exist that are contrary to expectations and these may prompt deeper investigation into the ecology of the study system. For example, all of the California sea lions in our dataset were breeding females restricted to central place foraging and were correctly assigned as CPFs except one: this individual exhibited foraging trips an order of magnitude greater in distance than its conspecifics, and as a result was classified as a migrant (Table 2; see Additional file 3: Figure S5 for movement paths). This result could subsequently direct researchers to more closely examine the behavior and ecology driving this intraspecific variation in foraging patterns.

Finally, it is important to note that individuals may transition between syndromes seasonally or during different life stages. One such example (not analyzed here) is the Pacific salmon (*Oncorhynchus* spp.), which undertakes a one-time migration as juveniles [71]. Individuals can also experience seasonal transitions, such as male springbok that enter a highly territorial phase [72] or pelagic seabirds that become CPFs [73, 74] during their breeding season. These transitions can explain why some individuals within a species that have the same life history pattern may be categorized differently, or appear at the interface between two syndromes. For example, among African wild dogs, which have annual denning periods during which they are restricted to central place foraging [75], most were classified as CPFs while some were classified as territorial and one was nomadic (Table 2). These differences can reasonably be explained by the life history stage of an individual during data collection. We include an approach to quantify the degree of intermediacy between syndromes in Additional file 5. Developing methods for dividing an individual’s trajectory into constituent movement syndromes is an arena ripe for future research.

## Conclusions

Our findings suggest that a relatively simple set of metrics can be used to reveal movement syndromes across taxa, environments, and spatial scales. While our study species span multiple taxa, movement modes, and order of magnitudes in body size, future work should continue to evaluate the generality of our approach by applying it to additional taxa. In short, our quantitative classification scheme opens the way to further studies relating movement syndromes to ecological factors, as well as life history traits. This has important implications for current attempts to incorporate species traits into climate change predictions [76]. For example, the inclusion of coarse classifications of species’ movement capacities (permanent resident, short-distance migrant, and long-distance migrant) into species distribution models has been shown to improve predictions of the probability of range shifts in response to climate change [77]. The movement syndrome concept can also inform predictions in a number of other areas of ecological research. For example, movement syndromes can be applied to macroecology to test whether species-area relationships vary between syndromes, in parallel to how they are expected to vary among taxa or geographic regions [78]. Classifying organisms by movement syndrome can inform predictions regarding the spatial dynamics of invasive species and disease ecology [9, 10], as well as the spatial distribution of resources in an organism’s environment [79]. To our knowledge, this is the first attempt to summarize measures of animal movement into broad movement syndromes evident across diverse systems — a framework that enables the generation of new insights into multiple aspects of ecology.

## Additional files

Additional file 1: **Table S1.** Summary and sources of GPS data used in analyses. (PDF 82 kb)

Additional file 2: R code for simulations of idealized movement syndromes. (R 37 kb)

Additional file 3: Supporting Figures S1-S5. (PDF 695 kb)

Additional file 4: Sensitivity of results to lower temporal resolution of movement data. (PDF 196 kb)

Additional file 5: Approach for quantifying the degree of intermediacy among syndromes. (PDF 128 kb)

## Abbreviations

CPF: Central place forager
MNSD: Mean net squared displacement
PCA: Principal components analysis
RT: Residence time
RW: Random walk
T2R: Time to return
TAC: Turn angle correlation
VI: Volume of intersection
