# Supplementary material for: Suite of simple metrics reveals common movement syndromes across vertebrate taxa
Source: Mov Ecol. 2017 Jun 1;5:12. doi: 10.1186/s40462-017-0104-2 (PMC5452391; doi:10.1186/s40462-017-0104-2)
Supplement: Supplementary file 1 — Table S1. Summary and sources of GPS data used in analyses. (PDF 82 kb) [file 40462_2017_104_MOESM1_ESM.pdf]

Table S1. Summary and sources of GPS data used in analyses.

Species	Unit Type	N individuals	Duration (mo.)	Fix interval pre-processing	Fix interval post-processing	Total fixes post-processing	Source
African buffalo	GPS	5	5 ± 2.2	1-hour	1-hour	11197	Bar-David *et al.* 2009
African elephant	GPS	8	17.5 ± 5.2	20-minute	1-hour	93583	Unpublished[3]
African wild dog	GPS	13 (1 per pack)	7 ± 2.3	5-minute	1-hour	55675	Unpublished[2], but see Abrahms *et al.* 2016
Black-backed jackal	GPS	15	9.4 ± 4.7	1-hour	1-hour	98678	Bellan *et al.* 2012
California sea lion	ARGOS	15	2.4 ± 0.3	variable	1-hour	26416	Unpublished[1]
Cheetah	GPS	5	7 ± 3.6	5-minute	1-hour	25421	Unpublished[2], but see Wilson *et al.* 2013
Galapagos albatross	GPS	8	3 ± 0.9	1.5-hour	1-hour	16979	Dodge *et al.* 2013
Galapagos tortoise	GPS	8	18 ± 2.7	1-hour	1-hour	103621	Blake *et al.* 2012
Lion	GPS	9	7 ± 3.3	5-minute	1-hour	41278	Unpublished[2]
N. elephant seal	ARGOS	15	7.5 ± 0.5	variable	1-hour	64281	Robinson *et al.* 2012
Springbok	GPS	10	8.7 ± 2.3	1-hour	1-hour	59593	Lyons, Turner & Getz 2013
White-backed vulture	GPS	10	8.5 ± 4.9	10-minute	1-hour	30454	Spiegel, Getz & Nathan 2013
Plains zebra	GPS	9	11.8 ± 3.1	20-minute	1-hour	67829	Unpublished[3]

[1]Daniel P. Costa, Department of Ecology and Evolutionary Biology, University of California-Santa Cruz, costa@ucsc.edu
[2]Alan M. Wilson, Structure & Motion Lab, Royal Veterinary College, University of London, awilson@rvc.ac.uk
[3]Wayne Getz, Department of Environmental Science, Policy, and Management, University of California-Berkeley, wgetz@berkeley.edu

Citations

Abrahms B, Jordan NR, Golabek KA, McNutt JW, Wilson AM, Brashares JS. Lessons from integrating behaviour and resource selection: activity-specific responses of African wild dogs to roads. Animal Conservation. 2016;19:247–55.

Bar-David S, Bar-David I, Cross PC, Ryan SJ, Knechtel CU, Getz WM. Methods for assessing movement path recursion with application to African buffalo in South Africa. Ecology. 2009;:1–13.

Bellan SE, Cizauskas CA, Miyen J, Ebersohn K, Küsters M, Prager KC, et al. Black-backed jackal exposure to rabies virus, canine

distemper virus, and *Bacillus anthracis* in Etosha National Park, Namibia. Journal of Wildlife Diseases. 2012;48:371–81.

Blake S, Yackulic CB, Cabrera F, Tapia W, Gibbs JP, Kümmeth F, et al. Vegetation dynamics drive segregation by body size in Galapagos tortoises migrating across altitudinal gradients. Fryxell J, editor. J Anim Ecol. 2012;82:310–21.

Dodge S, Bohrer G, Weinzierl R, Davidson SC, Kays R, Douglas D, et al. The environmental-data automated track annotation (Env-DATA) system: linking animal tracks with environmental data. Mov Ecol. BioMed Central; 2013;1:3.

Lyons AJ, Turner WC, Getz WM. Home range plus: a space-time characterization of movement over real landscapes. Mov Ecol. 2013;1:1–14.

Robinson PW, Costa DP, Crocker DE, Gallo-Reynoso JP, Champagne CD, Fowler MA, et al. Foraging Behavior and Success of a Mesopelagic Predator in the Northeast Pacific Ocean: Insights from a Data-Rich Species, the Northern Elephant Seal. Klimley AP, editor. PLoS ONE. 2012;7:e36728–13.

Spiegel O, Getz WM, Nathan R. Factors Influencing Foraging Search Efficiency: Why Do Scarce Lappet-Faced Vultures Outperform Ubiquitous White-Backed Vultures? Am Nat. 2013;181:E102–15.

Wilson AM, Lowe JC, Roskilly K, Hudson PE, Golabek KA, McNutt JW. Locomotion dynamics of hunting in wild cheetahs. Nature. Nature Publishing Group; 2013;498:185–9.
