# Supplementary material for: Suite of simple metrics reveals common movement syndromes across vertebrate taxa
Source: Mov Ecol. 2017 Jun 1;5:12. doi: 10.1186/s40462-017-0104-2 (PMC5452391; doi:10.1186/s40462-017-0104-2)
Supplement: Supplementary file 3 — Supporting Figures S1-S5. (PDF 695 kb) [file 40462_2017_104_MOESM3_ESM.pdf]

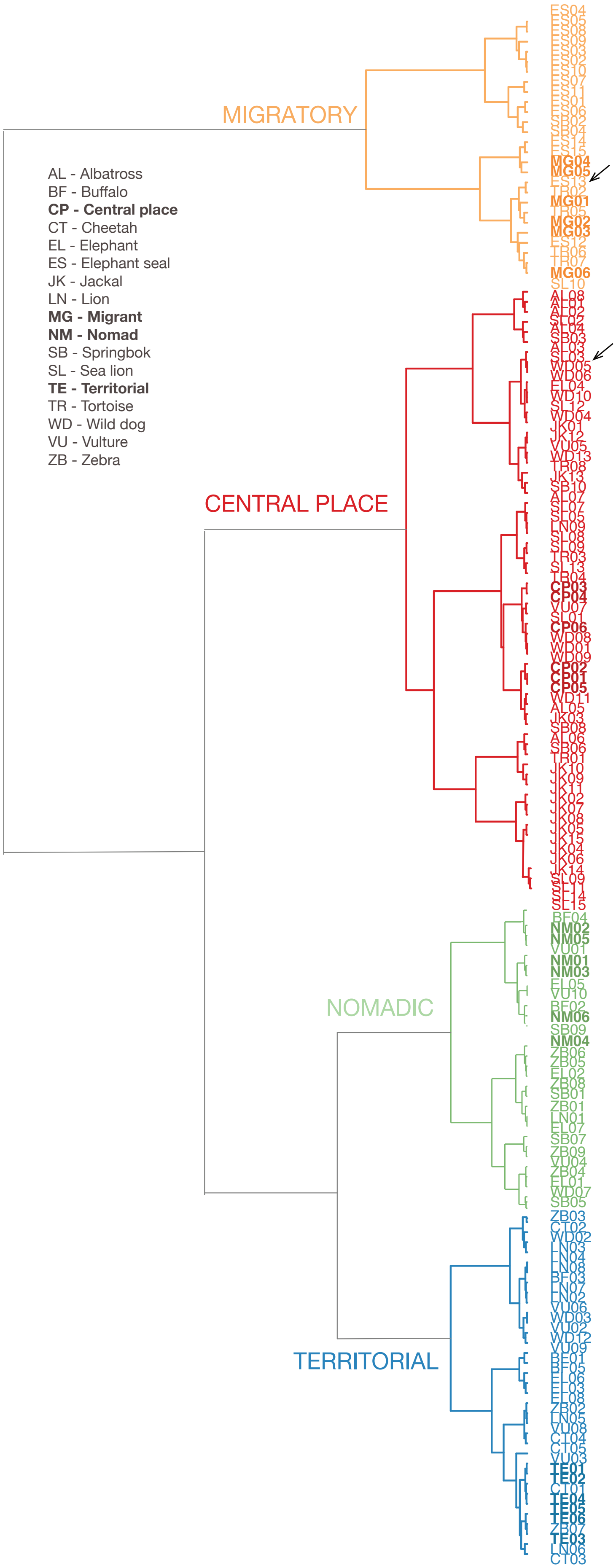

Figure S1. Full dendrogram displaying results of Ward hierarchical cluster analysis of all individuals based on PC1 and PC2 values. All individuals organize into four clusters, which can be considered movement syndromes. Simulated individuals are highlighted in bold. Two individuals (ES13 and WD05) are called out with arrows for illustration of syndrome intermediacy discussed in Additional File 5.

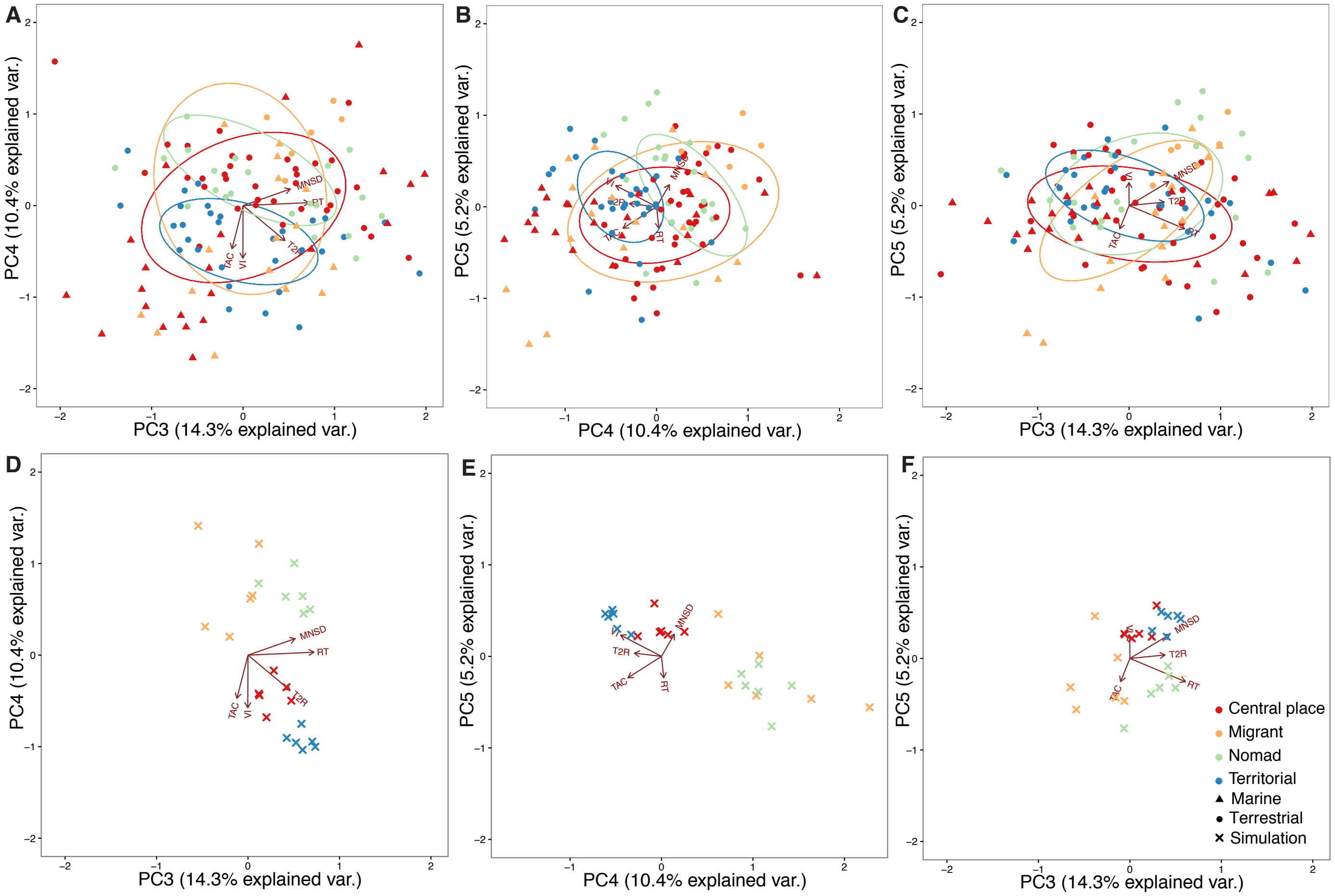

Figure S2. Scatterplot of classified empirical (panels A-C) and simulated (panels D-F) individuals based on minor PC axes (PCs 3, 4, and 5). Ellipses represent the 50% probability contour for cluster classifications based on PC1 and PC2.

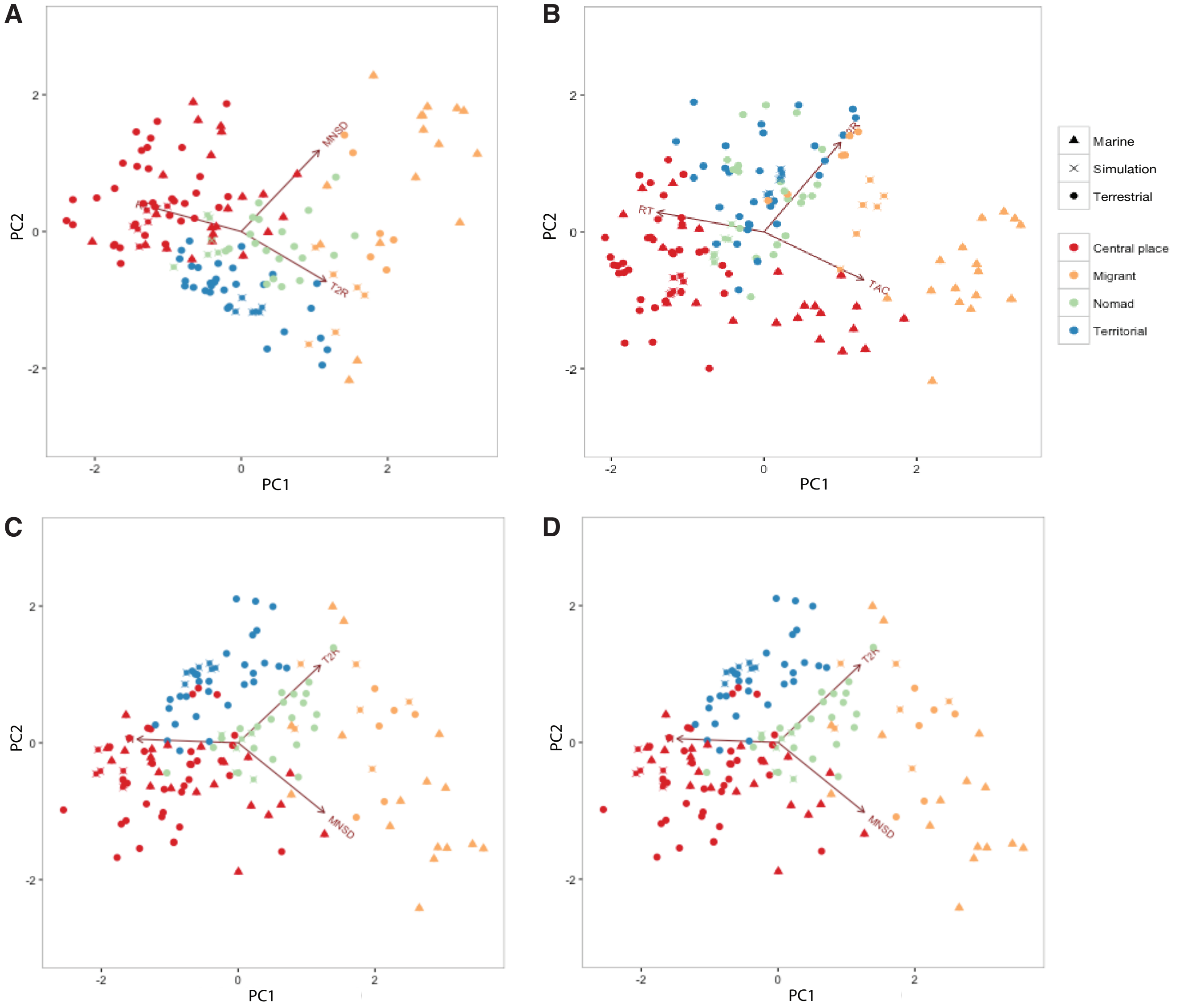

Figure S3. Scatterplots of classified individuals in PCA-defined axes based on a reduced set of three metrics to explore effects of collinearity between metrics: A) Residence Time (RT), Time-to-Return (T2R), and Maximum Net Squared Displacement (MNSD); B) RT, T2R, and Turn Angle Correlation (TAC); C) Volume of Intersection (VI), T2R, MNSD; and D) VI, T2R, and MNSD. Colors refer to cluster classifications based on the full set of five metrics for comparison.

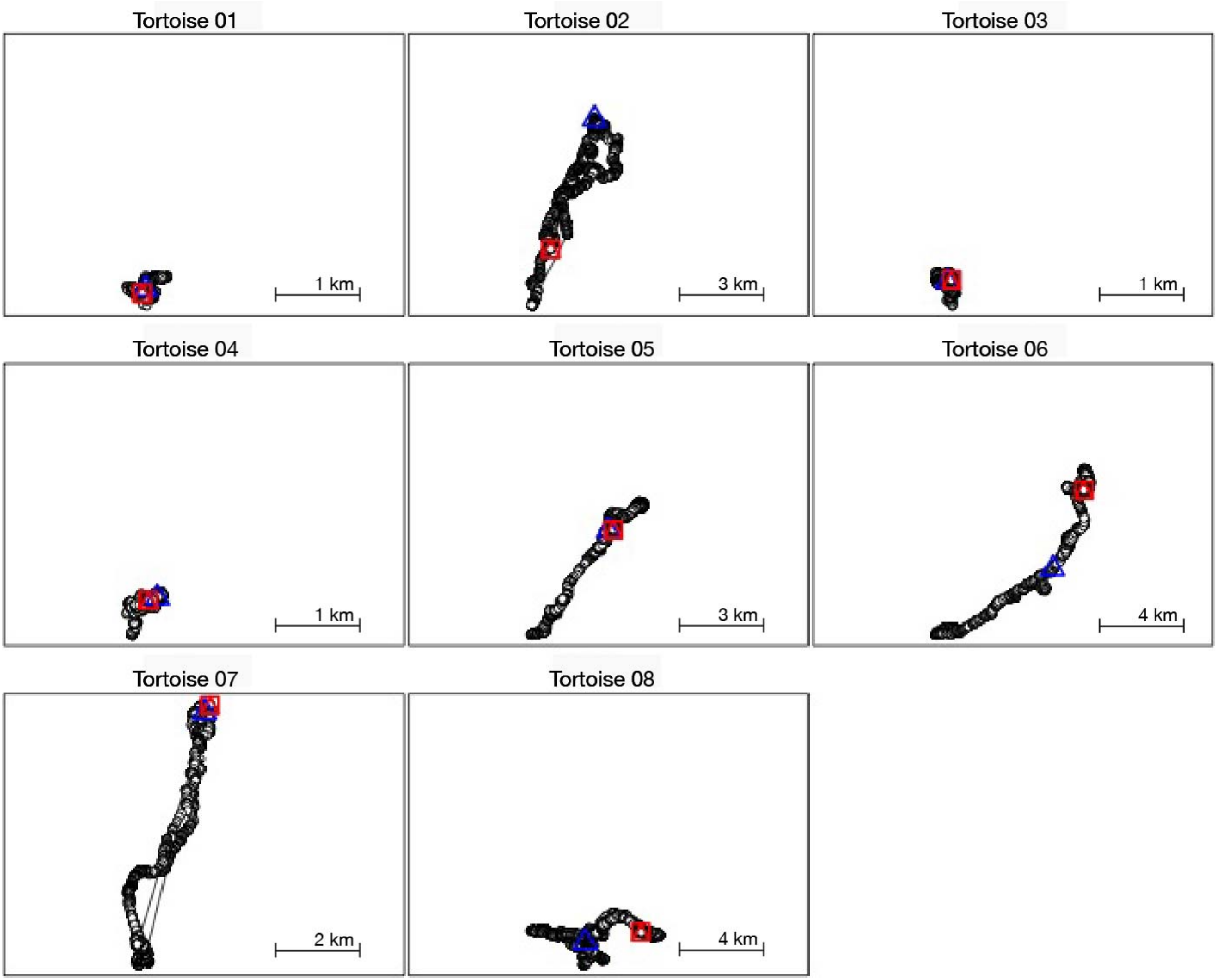

Figure S4. Movement paths of GPS-tracked resident (Tortoise 01, 03, 04, 08) and migratory Galapagos tortoises (Tortoise 02, 05-07). Movement paths begin at the blue triangle and end at the red square.

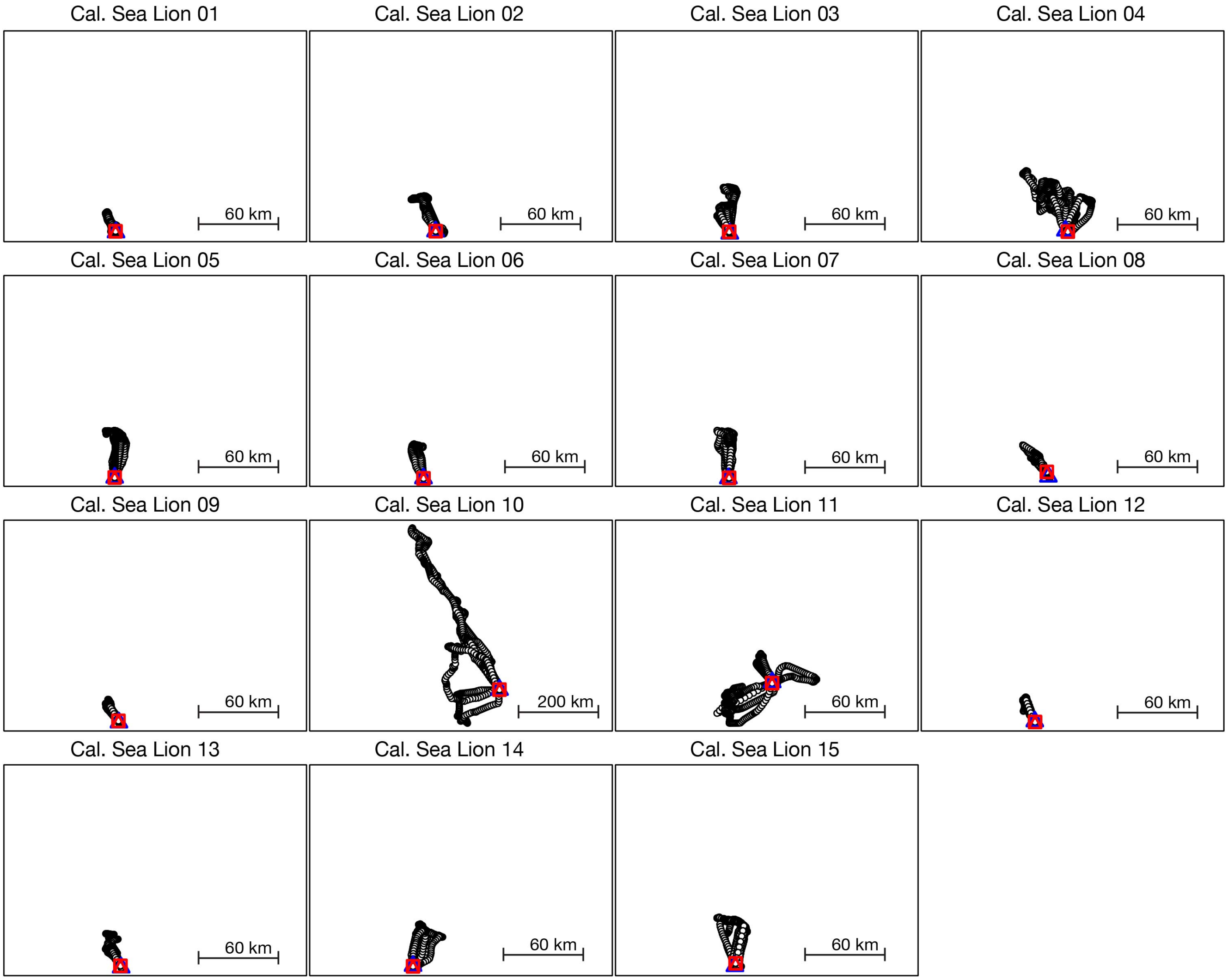

Figure S5. Movement paths of GPS-tracked California sea lions. Data were collected from females over a 3-month breeding period between November-February during which they exhibited central place foraging from the breeding colony. All individuals were appropriately classified as central place foragers except Sea Lion 10, which was classified as a migrant. Movement paths begin at the blue triangle and end at the red square.
