# Supplementary material for: Suite of simple metrics reveals common movement syndromes across vertebrate taxa
Source: Mov Ecol. 2017 Jun 1;5:12. doi: 10.1186/s40462-017-0104-2 (PMC5452391; doi:10.1186/s40462-017-0104-2)
Supplement: Supplementary file 4 — Sensitivity of results to lower temporal resolution of movement data. (PDF 196 kb) [file 40462_2017_104_MOESM4_ESM.pdf]

Sensitivity of results to lower temporal resolution of movement data

Table S2. Contributions of variables to and cumulative percentage of variance explained by principal components for movement data subsampled to 3-hour fix intervals.

	PC1	PC2	PC3	PC4	PC5
Turn Angle Correlation	0.48	0.31	-0.20	-0.71	0.36
Residence Time	-0.47	0.39	0.54	-0.00	0.57
Time-to-Return	0.33	-0.50	0.74	-0.25	0.09
Volume of Intersection	-0.54	0.12	0.04	-0.61	-0.56
Maximum Net Squared Displacement	0.38	0.69	0.32	0.24	-0.46
**Cumulative Percentage of Variance Explained**	**48.7%**	**70.7%**	**81.9%**	**93.4%**	**100%**

Table S3. Summary of 130 individuals within 13 species analyzed into cluster classifications for movement data at hourly intervals and subsampled to 3-hour fix intervals.

Species	***N ind.***	**Mig. (1-hr)**	**Mig. (3-hr)**	**CPF (1-hr)**	**CPF (3-hr)**	**Nomad. (1-hr)**	**Nomad. (3-hr)**	**Ter. (1-hr)**	**Ter. (3-hr)**
African buffalo	5	-	-	-	-	2	3	3	2
African elephant	8	-	-	1	-	4	1	3	7
African wild dog	13	-	-	9	7	1	1	3	5
Black-backed jackal	15	-	-	15	15	-	-	-	-
California sea lion	15	1	1	14	5	-	9	-	-
Cheetah	5	-	-	-	-	-	-	5	5
Galapagos albatross	8	-	-	8	6	-	2	-	-
Galapagos tortoise	8	4	4	4	4	-	-	-	-
Lion	9	-	-	1	2	1	3	7	4
N. elephant seal	15	15	15	-	-	-	-	-	-
Plains zebra	9	-	-	-	-	6	3	3	6
Springbok	10	2	-	4	5	4	5	-	-
White-backed vulture	10	-	-	2	7	3	2	5	1
**TOTAL**	130	22	20	58	51	21	29	29	30

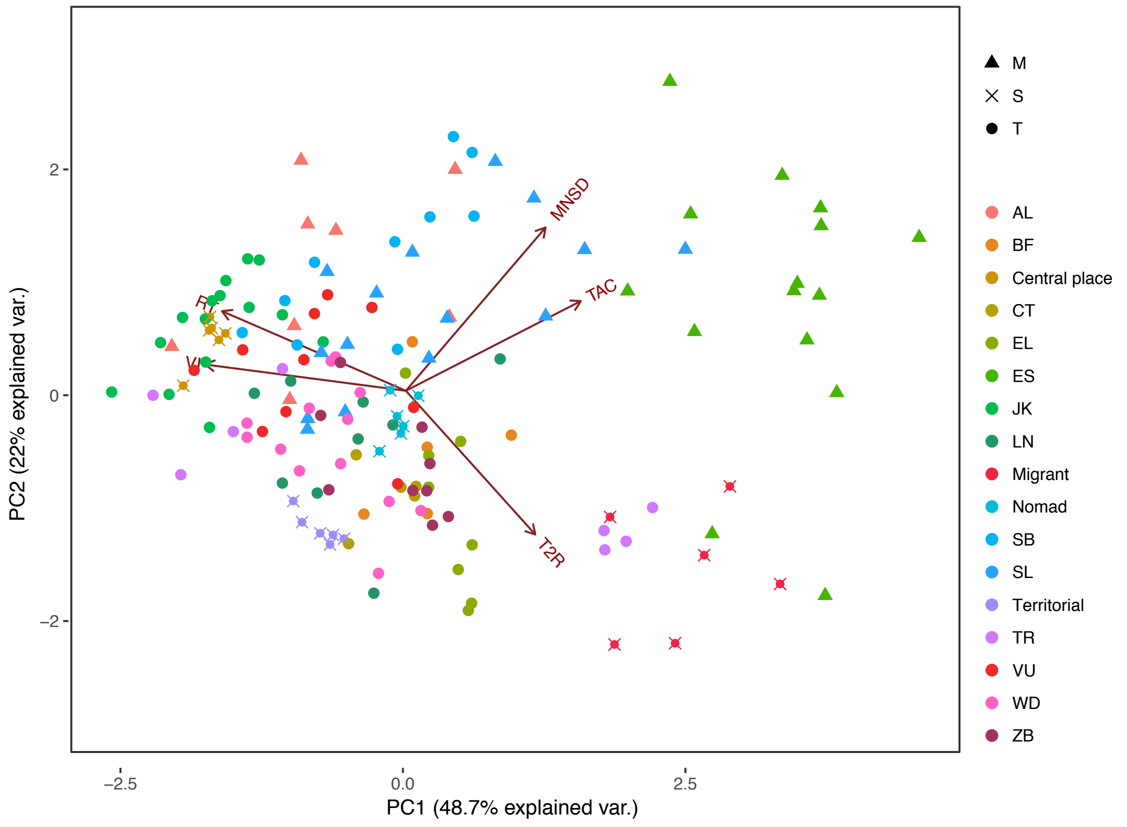

MNSD
TAC
T2R
RI
V
2
0
−2
−2.5
0.0
2.5
PC1 (48.7% explained var.)
PC2 (22% explained var.)
M
S
T
AL
BF
Central place
CT
EL
ES
JK
LN
Migrant
Nomad
SB
SL
Territorial
TR
VU
WD
ZB

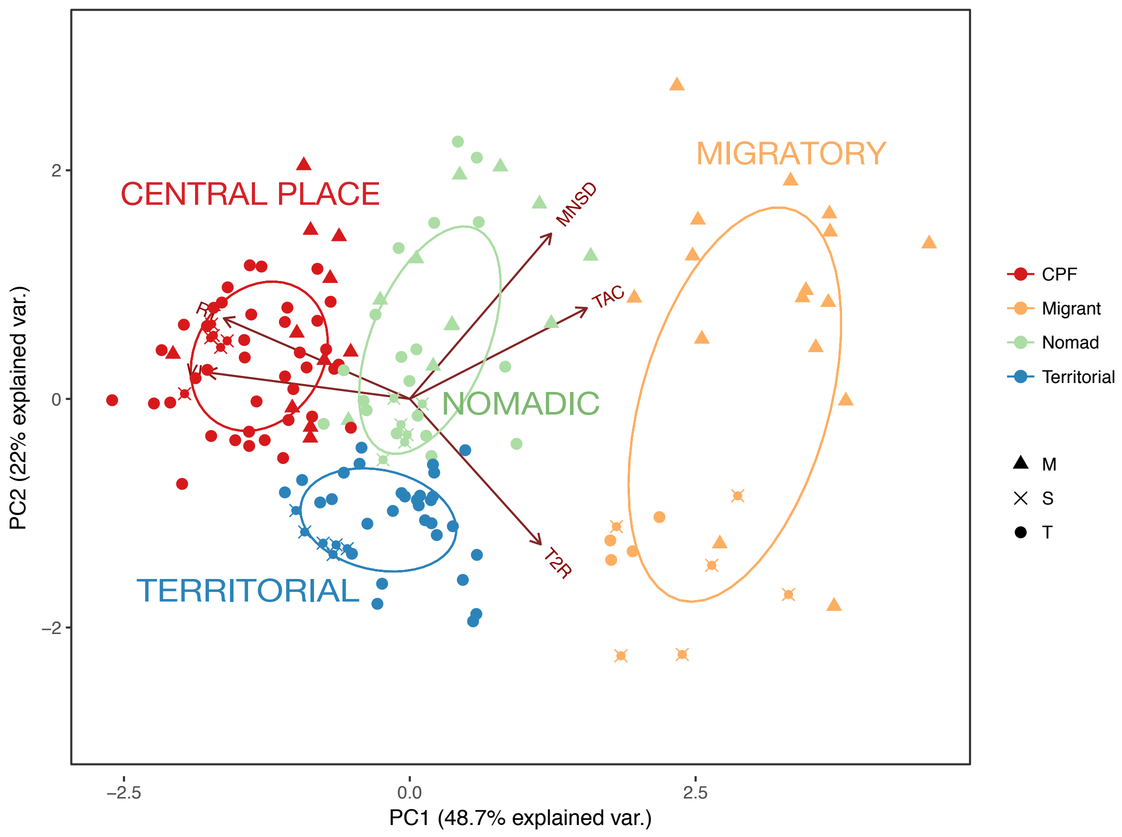
CENTRAL PLACE
MIGRATORY
NOMADIC
TERRITORIAL
MNSD
TAC
T2R
PC2 (22% explained var.)
PC1 (48.7% explained var.)
2
0
−2
−2.5
0.0
2.5
CPF
Migrant
Nomad
Territorial
M
S
T

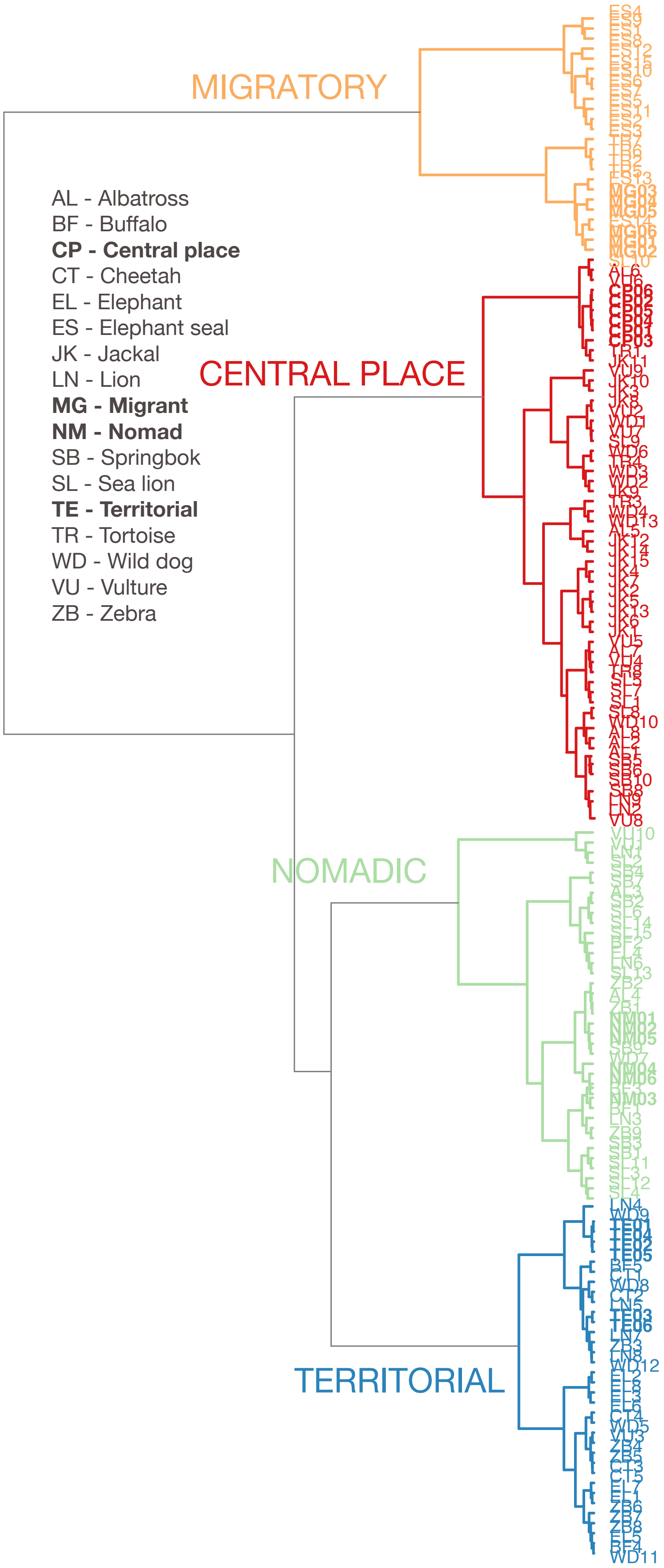
MIGRATORY
CENTRAL PLACE
NOMADIC
TERRITORIAL
AL - Albatross
BF - Buffalo
CP - Central place
CT - Cheetah
EL - Elephant
ES - Elephant seal
JK - Jackal
LN - Lion
MG - Migrant
NM - Nomad
SB - Springbok
SL - Sea lion
TE - Territorial
TR - Tortoise
WD - Wild dog
VU - Vulture
ZB - Zebra
