# Supplementary material for: Suite of simple metrics reveals common movement syndromes across vertebrate taxa
Source: Mov Ecol. 2017 Jun 1;5:12. doi: 10.1186/s40462-017-0104-2 (PMC5452391; doi:10.1186/s40462-017-0104-2)
Supplement: Supplementary file 5 — Approach for quantifying the degree of intermediacy among syndromes. (PDF 128 kb) [file 40462_2017_104_MOESM5_ESM.pdf]

Approach for quantifying the degree of intermediacy among syndromes

For any given individual movement trajectory, our approach provides a method for quantifying the degree of intermediacy among syndromes, akin to fractional trophic levels in community ecology (Pauly *et al.* 1998). This quantification is achieved by comparing the relative distance measured in terms of node heights within the dendrogram where the trajectory diverges from two different syndrome clusters. The “degree of intermediacy” can be defined in terms of ratios of the distances among neighboring clusters (measured by going ‘down’ or ‘up and then down’ the relevant node heights, as illustrated in Fig. S6). For purposes of discussion, we define the lowest node that includes all the simulated cases from one syndrome as the “strict cluster node.” If a particular trajectory is contained within this “strict syndrome cluster” then, following phylogenetic practices, we classify it as being that syndrome *sensu stricto*. On the other hand, if a particular trajectory falls outside the strict syndrome cluster but within the greater syndrome cluster (cf. Fig. S6), then we classify this trajectory as being the syndrome *sensu lato*. In this case, we can take the further step of calculating the relative distance of that trajectory’s node to its defining *sensu stricto* cluster node compared with its distance to another *sensu stricto* cluster nodes. To illustrate using our dendrogram in Fig. S1, the trajectory of Elephant Seal #13 (ES13) is migrant (MG) *sensu stricto*. In contrast, African Wild Dog #5 (WD05) is CPF *sensu lato*, but because its distance to the CP node is roughly 4 and to the strict territorial (TE), nomad (NM), and migrant (MG) nodes are roughly 9, 9 and 17 respectively, we can make statements such as, this individual’s trajectory is 9/4=2.25 times more CPF-like than TE- or MN-like, and 17/4=4.25 times more CPF- than MG-like.

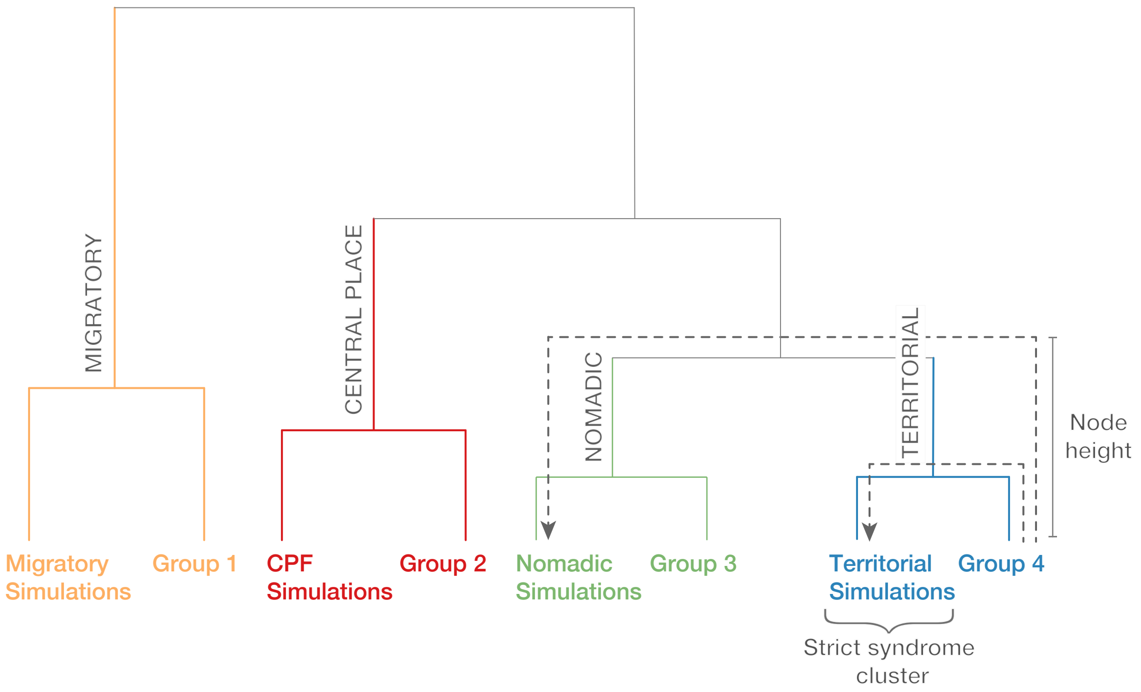

Figure S6. Illustration of method for quantifying the degree of intermediacy among movement syndromes for a given trajectory in a hypothetical dendrogram. The "strict syndrome cluster" is defined as the lowest cluster that contains all the simulations from a single syndrome. If a given trajectory is contained within the strict syndrome cluster then it is classified as being that syndrome *sensu stricto*. If a trajectory falls outside the strict syndrome cluster but within the greater syndrome cluster, then it is classified as being that syndrome *sensu lato*. In this diagram, a trajectory in Group 4 would be considered Territorial in *sensu lato.* Its degree of intermediacy between Territorial and Nomadic syndromes would be the ratio between its distance in terms of node heights to the strict Nomadic syndrome cluster and its distance to the strict Territorial syndrome cluster (gray hashed arrows).
